# Preventing cholera outbreaks through early targeted interventions

**DOI:** 10.1371/journal.pmed.1002510

**Published:** 2018-02-27

**Authors:** Lorenz von Seidlein, Jacqueline L. Deen

**Affiliations:** 1 Mahidol-Oxford Tropical Medicine Research Unit (MORU), Faculty of Tropical Medicine, Mahidol University, Bangkok, Thailand; 2 Centre for Tropical Medicine and Global Health, Nuffield Department of Medicine, University of Oxford, Oxford, United Kingdom; 3 Institute of Child Health and Human Development, National Institutes of Health, University of the Philippines, Manila, Philippines

## Abstract

In a Perspective, Lorenz von Seidlein and Jacqueline L. Deen discuss the implications of Andrew Azman and colleagues' accompanying study for management of cholera outbreaks.

The technology for a killed oral cholera vaccine (OCV) was developed by Jan Holmgren and colleagues in Sweden in the 1970s and 1980s [1] and, following clinical evaluation, was acquired by vaccine producers in developing countries, including Vietnam and India. One resulting low-cost OCV, Shanchol, received WHO prequalification in 2011 [2], which allowed the vaccine to be purchased by United Nations agencies [3]. For many years, however, WHO did not recommend oral cholera vaccination once an outbreak had started. But in October 2009, the WHO Strategic Advisory Group of Experts (SAGE) on immunization made the pivotal recommendation that oral cholera vaccination should be considered as a reactive strategy in areas with ongoing outbreaks [4].

The prequalification of Shanchol coincided with the massive cholera outbreak in Haiti that started after the 2010 earthquake. Despite the updated SAGE recommendation, the Pan American Health Organisation (PAHO) advised against the use of OCVs in Haiti, citing “limited vaccine availability, complex logistical and operational challenges of a multidose regimen, and obstacles to conducting a campaign in a setting with population displacement and civil unrest” [5,6]. More than a year after the start of the outbreak, nongovernmental organizations (NGOs) started to use OCVs in Haiti, but cholera had already become endemic throughout the country [7,8]. The Haitian cholera outbreak has resulted in over 800,000 cholera cases and 9,480 deaths, a large proportion of which could arguably have been prevented by early vaccination using OCV. Especially during complex humanitarian disasters when public health infrastructures are overloaded and the restoration of water supply and sanitation is years away, it is now recognised that oral cholera vaccination can prevent cholera and suppress outbreaks until more permanent solutions can be established. In this issue of *PLOS Medicine*, Andrew Azman and colleagues extend this important concept by studying the possible impact of various targeted interventions, including cholera vaccination, in cholera outbreaks [9].

## Demand for cholera vaccine outstrips supply

The establishment of an OCV stockpile by WHO and its partners, in 2013, has led to a larger vaccine supply [10]. The stockpile is primarily for emergency response, to reduce the extent of cholera outbreaks or prevent their occurrence in the context of humanitarian crises. When Gavi, the Vaccine Alliance, agreed to provide funding for OCVs in November 2013, it became possible to roll out the vaccine on a larger scale [11]. A second prequalified killed OCV, Euvichol, became available in 2016 [10,12]. The current global production capacity for OCVs has been estimated at over 25 million doses per year [13]. Yet to the surprise of many, the demand for OCVs continues to outstrip supply. The increased need for OCV appears to correlate with the improved reporting of cholera outbreaks. Before OCVs became available for mass vaccination campaigns, countries had more to lose than gain from reporting cholera outbreaks; in particular, the tourism and seafood export sectors suffer once an outbreak is declared. Since an effective cholera vaccine stockpile is now available, it appears that countries are more open to acknowledging outbreaks and requesting vaccine.

## Cholera outbreaks: Hit or miss

With funding from Gavi in place and an increasing OCV supply assured, one would think that large and disastrous cholera outbreaks would have been confined to history. However, events during the past few months have demonstrated how much of a hit-or-miss affair cholera outbreak control and prevention remains. In October 2017, 900,000 doses of OCV were mobilised from the international stockpile to prevent cholera outbreaks in Rohingya camps along the border between Bangladesh and Myanmar, even before cholera cases were reported [14]. Considering the cholera endemicity in Bangladesh in combination with desperate conditions in the refugee camps, WHO and the stockpile managers are to be congratulated for their timely action and foresight.

In contrast, OCV doses did not arrive in Yemen despite a humanitarian crisis of enormous proportions (994,751 suspected cases; 2,226 deaths by December 2017) [15]. Not only did WHO fail to mount the essential mass vaccination campaigns, but the explanation for this omission was once more that oral cholera vaccinations during an ongoing outbreak are inappropriate [16], a statement that is factually incorrect. Management of the Yemen cholera outbreak has been complex, and many parties carry responsibility for the lack of successful interventions. Yet, WHO has the mandate to support decision-making in such difficult circumstances. By the time the vaccines were requested and shipment was finally approved (though never used), the outbreak had already reached its peak, highlighting the critical need for a rapid response.

The decision of which requests to the OCV stockpile should be granted (and for how many doses) falls to an International Coordinating Group (ICG) composed of representatives from United Nations Children's Fund (UNICEF), Médecins Sans Frontières (MSF), the International Federation of Red Cross and Red Crescent Societies (IFRC), and WHO [17]. Over the years, some requests were rejected on technical grounds, while others were assigned too low a priority to ever be delivered. Ultimately, only half the requested doses (51%; 12.8 million doses) have been shipped in 46 deployments between 2013 and 2017 [10]. The criteria for these decisions and any competing interests of the coordinating group members are not transparently disclosed, and the decisions do not always appear fair; yet they have far-reaching consequences.

## A new strategy for OCV use?

The public health strategy for oral cholera vaccination has broadened—from purely preemptive use in mitigating cholera outbreaks to the control of endemic cholera in conjunction with other prevention measures, such as water, sanitation, and hygiene interventions and social mobilization. The underlying dilemma in decision-making on OCV deployment is the inability to predict where and when a handful of cholera cases will blow up into a massive outbreak or whether small outbreaks will fizzle out without any further intervention. Once a massive cholera outbreak paralyses a population, it is easy to agree on the need for mass vaccination campaigns. But during nascent outbreaks with large margins of uncertainty, public health officials are reluctant to allocate substantial resources for mass vaccination campaigns, and the ICG is reluctant to release the limited global stock. Yet, early intervention can prevent a public health catastrophe.

This is where the targeted approach proposed by Azman and colleagues in *PLOS Medicine* comes into play [9]. The authors modelled the impact of case-area targeted interventions (CATIs), which can include improved water quality and supply, sanitation, handwashing, OCV, and prophylactic antibiotics. The authors based their study on parameters from a cholera outbreak in N'Djamena, Chad, in 2011 and simulated the impact of targeted interventions in a variety of potential epidemics. They estimate that vaccinating people within 100 m around index case households and improving their water source early in epidemics would reduce the number of cases by 82% (IQR 71 to 88) compared to uncontrolled epidemics. The simulated additional antibiotic treatment of neighbours within a 30-m to 45-m radius around the index case was helpful, but only in the short term.

This targeted approach not only should be less resource intensive than mass drug administration, but it also lends itself to speedy implementation. In combination with appropriate surveillance, the key decision makers should be able to see whether the outbreak is under control. When cholera incidence is stable, the targeted intervention should be continued until no more cases are reported. But if there is an increase in cases, targeted interventions should be expanded to mass vaccination campaigns. CATI could allow the local response team to acquire experience with the distribution of OCV, the local population to see that OCVs are safe, and the ICG to be assured that the vaccines are needed and are in reliable hands.

As with all mathematical models, there is a need for empirical confirmation of how well Azman and colleagues’ intervention performs and if it is even feasible. For the CATI strategy to be successful, cholera cases have to be detected quickly, sufficient OCV doses must be available on site within a short time of detection of the first cases, and the logistics for contact tracing and vaccination have to be set up immediately. Moreover, the cost and feasibility of integrating a sustainable surveillance and response system into a government’s health infrastructure has yet to be assessed.

## Speeding up implementation in cholera outbreaks

In October 2017, a WHO-appointed Global Task Force on Cholera Control [18] convened a meeting that called for the ambitious goal of ending cholera outbreaks within the next decade [13]. Targeted interventions, if proven effective, could help achieve this goal. Instead of waiting for requests, the managers of the stockpile could monitor the global cholera situation, recommend early targeted interventions, and simultaneously clear the hurdles for the shipment of vaccines. Such a proactive approach would reduce the time between reporting of the first cases and the first vaccination. The success of the targeted approach would have to be closely monitored, and, if not successful, OCV shipments and mass vaccination campaigns must follow without delay. Vietnam has for all practical purposes managed to eliminate cholera using an integrated approach that included targeted OCV deployment [19]. Other countries should have the tools to do the same.

## Abbreviations

CATI: case-area targeted intervention
ICG: International Coordinating Group
IFRC: International Federation of Red Cross and Red Crescent Societies
MSF: Médecins Sans Frontières
NGO: nongovernmental organization
OCV: oral cholera vaccine
PAHO: Pan American Health Organisation
SAGE: Strategic Advisory Group of Experts
UNICEF: United Nations Children's Fund
